# Targeting an inflammation-amplifying cell population can attenuate osteoarthritis-associated pain

**DOI:** 10.1186/s13075-024-03284-y

**Published:** 2024-02-17

**Authors:** Akshay Pandey, Mamta Singla, Ana Geller, Stuart B. Goodman, Nidhi Bhutani

**Affiliations:** Department of Orthopaedic Surgery, Stanford School of Medicine, 240, Pasteur Drive, Biomedical Innovations Bldg, Stanford, CA 94034 USA

**Keywords:** Osteoarthritis, Pain, Senescence, Inflammation

## Abstract

**Background:**

Understanding of pain in osteoarthritis, its genesis, and perception is still in its early stages. Identification of precise ligand-receptor pairs that transduce pain and the cells and tissues in which they reside will elucidate new therapeutic approaches for pain management. Our recent studies had identified an inflammation-amplifying (Inf-A) cell population that is expanded in human OA cartilage and is distinctive in the expression of both IL1R1 and TNF-R2 receptors and active Jnk signaling cascade.

**Methods:**

In this study, we have tested the function of the cartilage-resident IL1R1^+^TNF-R2^+^ Inf-A cells in OA. We have identified that the IL1R1^+^TNF-R2^+^ Inf-A cells expand in aged mice as well as after anterior cruciate ligament tear upon tibia loading and OA initiation in mice. We targeted and modulated the Jnk signaling cascade in InfA through competitive inhibition of Jnk signaling in mice and human OA explants and tested the effects on joint structure and gait in mice.

**Results:**

Modulation of Jnk signaling led to attenuation of inflammatory cytokines CCL2 and CCL7 without showing any structural improvements in the joint architecture. Interestingly, Jnk inhibition and lowered CCL2 and 7 are sufficient to significantly improve the gait parameters in treated PTOA mice demonstrating reduced OA-associated pain. Consistent with the mice data, treatment with JNK inhibitor did not improve human OA cartilage explants.

**Conclusion:**

These studies demonstrate that Inf-A, an articular-cartilage resident cell population, contributes to pain in OA via secretion of CCL2 and 7 and can be targeted via inhibition of Jnk signaling.

**Supplementary Information:**

The online version contains supplementary material available at 10.1186/s13075-024-03284-y.

## Background

Osteoarthritis (OA) is the most common form of age-associated arthritis that is characterized by progressive degeneration of joint tissues including cartilage and bone along with synovitis [1, 2]. It is a multifactorial disease that can be exacerbated by physical trauma, obesity, and systemic inflammation besides aging [3–6]. It is often diagnosed only in the later stages of disease with pain being the major cause for seeking medical attention; hence, the early events in OA initiation and pathogenesis are unclear [7]. A further complication is that the severity of pain and the structural damage in the joint may not always be correlated, with different rates being observed for the progression in radiographic damage and pain in patients [8, 9]. Due to these challenges, it has been extremely difficult to design an effective OA therapeutic and there are no existing disease-modifying drugs for OA (DMOAD) [10].

While pain in OA and the resulting limitation in activity or loss of mobility are the most relevant clinical symptoms that need to be addressed, the source of the pain is not well understood [1, 8, 10]. It has been observed that inflammatory pain is prevalent in most of the cases as compared to nociceptive or neuropathic pain associated with OA [11–13]. In the late stages of the disease, there is substantial damage to cartilage, bone, and meniscus along with synovitis hence the joint is a highly inflammatory environment [14]. Recent studies indicate that this local inflammation along with neuroinflammation and possibly systemic chronic inflammation are collectively responsible for the genesis of the complex OA pain [15–17]. An increased understanding of the molecular mediators of pain as well as the specific cellular populations that secrete these factors is required for devising targeted therapies for OA pain.

Recent advances in single-cell biology are leading to an increased understanding of distinct cell populations in joint tissues [18–21]. These studies have identified normal and pathogenic single-cell populations including rare populations like senescent cells that play a regulatory role in OA by secreting senescence-associated secretory proteins (SASP) to affect neighboring cells [22, 23]. Our recent studies have identified rare populations associated with OA cartilage that can modulate inflammation in the end-stage disease [20]. These include the inflammation-amplifying (Inf-A) population characterized by the expression of both IL1R1 and TNF-R2 receptors, and the inflammation-dampening (Inf-D) population characterized by CD24 expression. Inf-A population is unique in demonstrating active JNK signaling in human OA cartilage isolated from the joint tissues after total knee replacement of patients. The transcriptomic analysis of Inf-A cells from available scRNA-seq data sets revealed that innate and adaptive immune cell signaling, inflammation, and altered T and B cell signaling were highly enriched suggesting that Inf-A may play a role in recruiting immune cells to the joint space. Selective targeting of Inf-A cells through a JNK inhibitor decreased overall inflammation in OA chondrocytes, with a significant decrease in the levels of cytokines CCL2 and CCL7. While these observations in end-stage human OA cartilage identified a role for this pathogenic population, they did not provide information on how the single-cell landscape changes from a healthy state during disease progression [20]. In this study, we report on the timing of appearance and effects of the Inf-A population in OA pathogenesis. Furthermore, we elucidate a role for the Inf-A subpopulation in both aged mice and in a well-established mouse model of post-traumatic OA.

## Materials and methods

### Animal procedures

All animal procedures were approved by the Stanford University Administrative Panel on Laboratory Animal Care (APLAC 27507). Tibia loading was performed on 3–4-month-old male C57BL/6 mice per published methodology [24–27].

### Histology and immunohistochemistry

Lower limbs isolated from tibia-loaded mice were fixed in 10% neutral buffered formalin for 24 h and transferred into 70% ethanol. Further processing and sectioning were performed at Histoserv (Germantown, MD). Tissue sections were cut at 6-μm thickness with 50-μm space between consecutive sections. Cartilage tissue sections were stained for Safranin O and fast green or used. Immunohistochemistry for Jnk (CST, 9255S; 1:250) and p16 (Thermofisher, MA5-17,142; 1:250) was carried out.

Human cartilage from joint tissues after total knee replacement of patients (under Stanford-approved IRB protocols) was divided into smaller discs using biopsy punches (2-mm diameter). These cartilage discs were then cultured with or without JNK inhibitor for a week before processing. The tissues were embedded in OCT and sectioned in Leica cryotome at 6-μm thickness and stained as described above.

### Statistical analyses

Animal experiments involved at least three independent and randomly chosen mice at comparable developmental stages. The statistical differences were determined using Student’s *t* test with Welch’s correction, using GraphPad Prism 8.0 for Windows, unless otherwise stated. All values are expressed as means ± s.e.m. along with the number of individual mice/samples analyzed (*n*). *p* value of < 0.05 is accepted as statistically significant.

Please refer to Additional file 1: supplemental methods for detailed methodology.

## Results

### *IL1R1*^+^*TNF-R2*^+^*inflammation-amplifying (InfA) cells expand in aged and osteoarthritic cartilage in mice*

**Fig. 1:**
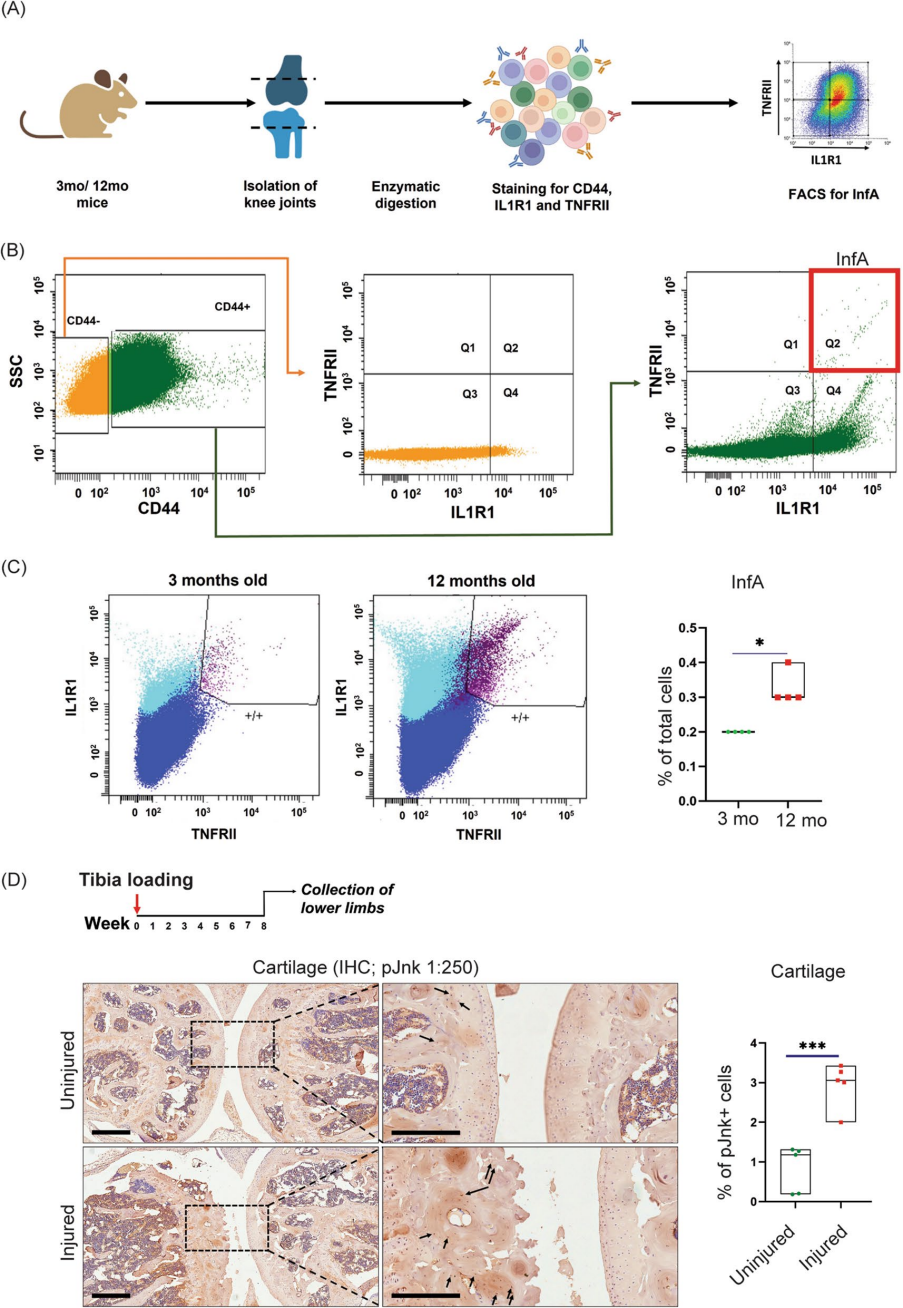
Inflammation-amplifying (InfA) cells expand in aged and osteoarthritic cartilage in mice. **A** Schematic showing strategy for isolation of cells of InfA cells from mice knee joints. **B** FACS strategy for isolation of TNF-R2 and IL1R1 double-positive InfA cells. **C** FACS plots showing InfA population in 3-mo and 12-month-old mice; cell numbers were quantified and are represented in the bar graph as a percentage of the total number of analyzed cells. **D** Schematic showing induction of OA using tibia loading model and collection of limbs for analysis at 8 weeks post-injury, representative sagittal sections of knee joint cartilage from tibia-loaded and control limbs showing the presence of p-Jnk + cells (InfA cells) in the cartilage, quantification in the bar graph on the right. Significance was calculated using Student’s *t* test with Welch’s correction. *p < 0.05, **p < 0.01, ***p < 0.001, scale bar = 200 μm (**D**)—left panel, scale bar = 100 μm (**D**)—right panel

Our previous studies demonstrated an important role for the IL1R1^+^TNF-R2^+^ inflammation-amplifying (InfA) cells in regulating the secretome of end-stage human OA chondrocytes [20]. In order to further investigate this population, we utilized mice to test whether the InfA cells expand with age or upon joint injury. Towards this goal, we utilized C57BL/6 mice aged 3 months (young adult) or 12 months (old). Knee joints from these mice were harvested followed by mechanical and enzymatic digestion with collagenase to isolate single cells. Cells were stained for CD44, a marker for chondrocytes along with TNF-R2 and IL1R1 (Fig. 1A). Only CD44^+^ cells in the young as well as aged mice showed the presence of the IL1R1^+^TNF-R2^+^ double-positive InfA population (Fig. 1B). These cells were absent in the CD44^−^ cells that were the non-cartilaginous cells in the joint presumably consisting of contaminating bone, synovium, or immune cells. Interestingly, there was a significant increase in the InfA cells in the old mice as compared to the 3-month-old mice (Fig. 1C).

To test whether such an age-associated expansion of the InfA population is also observed upon injury, we utilized a post-traumatic OA (PTOA) mouse model, wherein tibia loading leading to ACL rupture is the trigger to initiate OA (Fig. 1D). This PTOA model mimics ACL injuries in human patients and is widely utilized [24, 25, 28]. Lower limbs of the tibia-loaded mice were collected at 8 weeks post-injury when OA characteristics are easily observed and analyzed for the InfA population by immunohistochemistry. As our previously published data has demonstrated that only the InfA cells are characterized by active JNK signaling in OA cartilage, we have utilized p-Jnk staining to mark InfA cells. Consistent with the cartilage in aged mice, we found a significant increase in the number of p-Jnk expressing cells in the cartilage of the injured limbs of OA mice compared to the uninjured controls (Fig. 1D). We also found an increase in p-Jnk-expressing cells in the meniscus and synovium of these mice (Additional file 2: Figure S1A, B). These data established that similar to the end-stage human cartilage, there is an expansion of InfA cells in age- and injury-associated OA in mice.

### Modulation of Inf A through a Jnk inhibitor does not affect joint degeneration

**Fig. 2:**
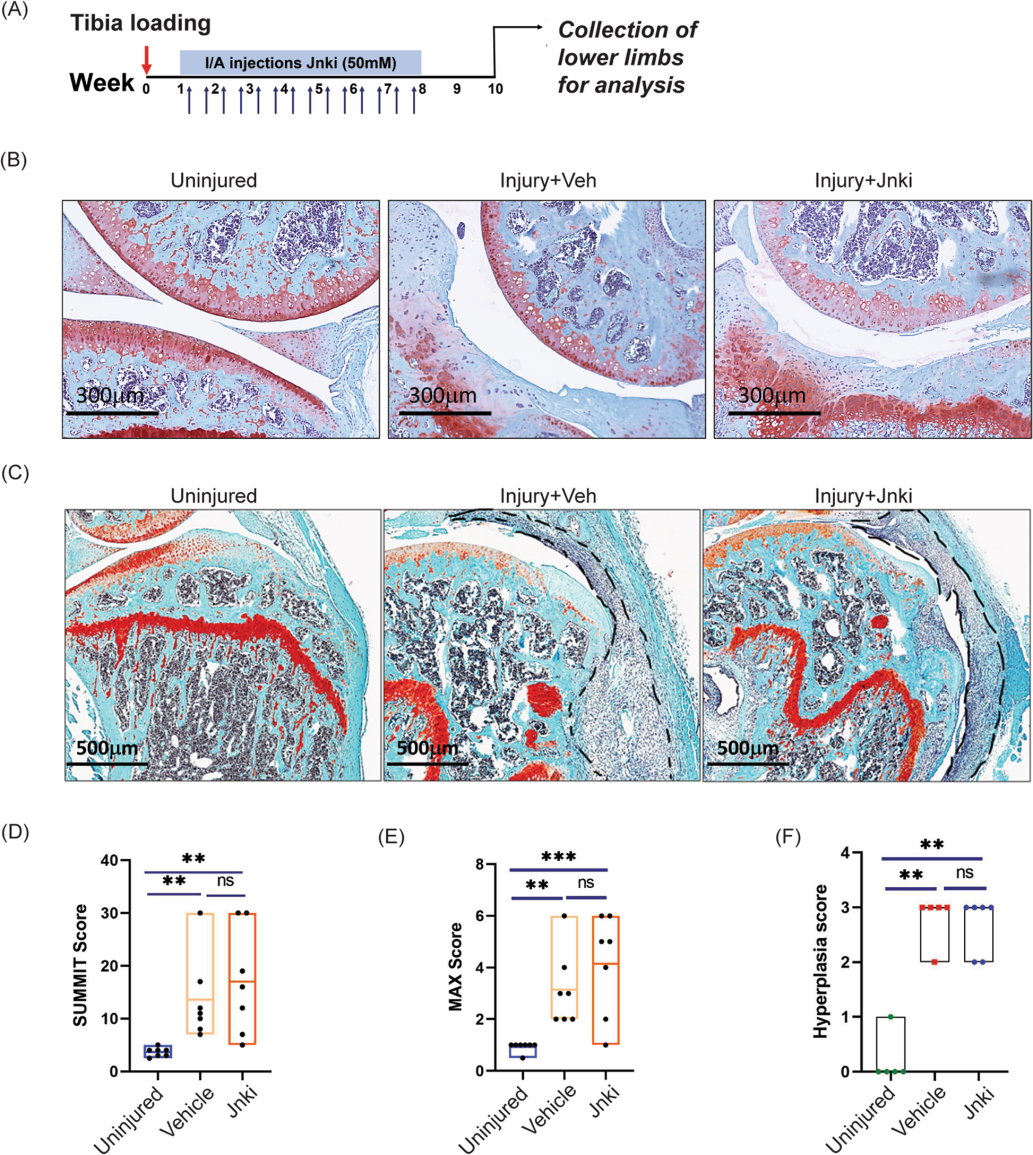
Modulation of Inf A through a Jnk inhibitor (Jnki) in the tibia loading model for PTOA. **A** Schematic showing strategy for Jnk inhibitor administration starting 1 week post-tibia loading. **B** Sagittal sections of knee joint showing cartilage degradation at 10 weeks upon tibia loading. **C** Sagittal sections showing inflamed synovium in the knee cartilage in the tibia-loaded mice. **D**, **E** OARSI scores for knee cartilage at 10 weeks post-tibia loading (*n* = 7). **F** Hyperplasia score for synovium lining in the tibia-loaded mice (Uninjured (*n* = 5), Vehicle (*n* = 5), Jnki (*n* = 6)). Significance was calculated using Student’s *t* test with Welch’s correction. *p < 0.05, **p < 0.01, ***p < 0.001

To investigate the role of the InfA cells in OA progression, we tested the effects of its inhibition in the PTOA model [24, 25, 28]. Towards this aim, we utilized a small molecule inhibitor against p-Jnk (SP600125, referred to as JNKi from hereon) to inhibit active JNK signaling in InfA in mice similar to our published data in human OA cartilage [29, 30]. The small molecule p-JNK inhibitor, Jnki (50 mM), or vehicle control was administered intra-articularly in mice (*n* = 7) twice a week starting at 1 week post-tibia loading (Fig. 2A). Administration of Jnki continued till 8 weeks after which mice were either tested for gait analysis or the joints were collected and analyzed (Fig. 2A). Proteoglycan staining with Safranin O (SafO) showed no protection against proteoglycan loss or articular cartilage erosion in the injured joints treated with JNKi (Fig. 2B). Grading of the loaded and unloaded joints using OARSI scoring [31] showed an increased score upon OA induction suggesting increased cartilage loss and erosion; however, there was no chondroprotective effect of JNKi. There was no statistical difference in the summit or max OARSI scores between vehicle or JNKi-treated joints (Fig. 2D, E), suggesting that JNKi has no effect on cartilage or joint structure preservation.

Furthermore, upon investigating synovial hyperplasia in the PTOA mice, synovial hyperplasia was significantly increased in the injured limbs as compared to the uninjured limbs as expected (Fig. 2C). However, synovial hyperplasia was unaffected in the injured PTOA limbs with the administration of Jnki, again demonstrating that JNKi does not protect against overall synovial infiltration (Fig. 2C, F).

### Modulation of InfA through a Jnk inhibitor attenuates inflammatory cytokines and OA-associated pain

**Fig. 3:**
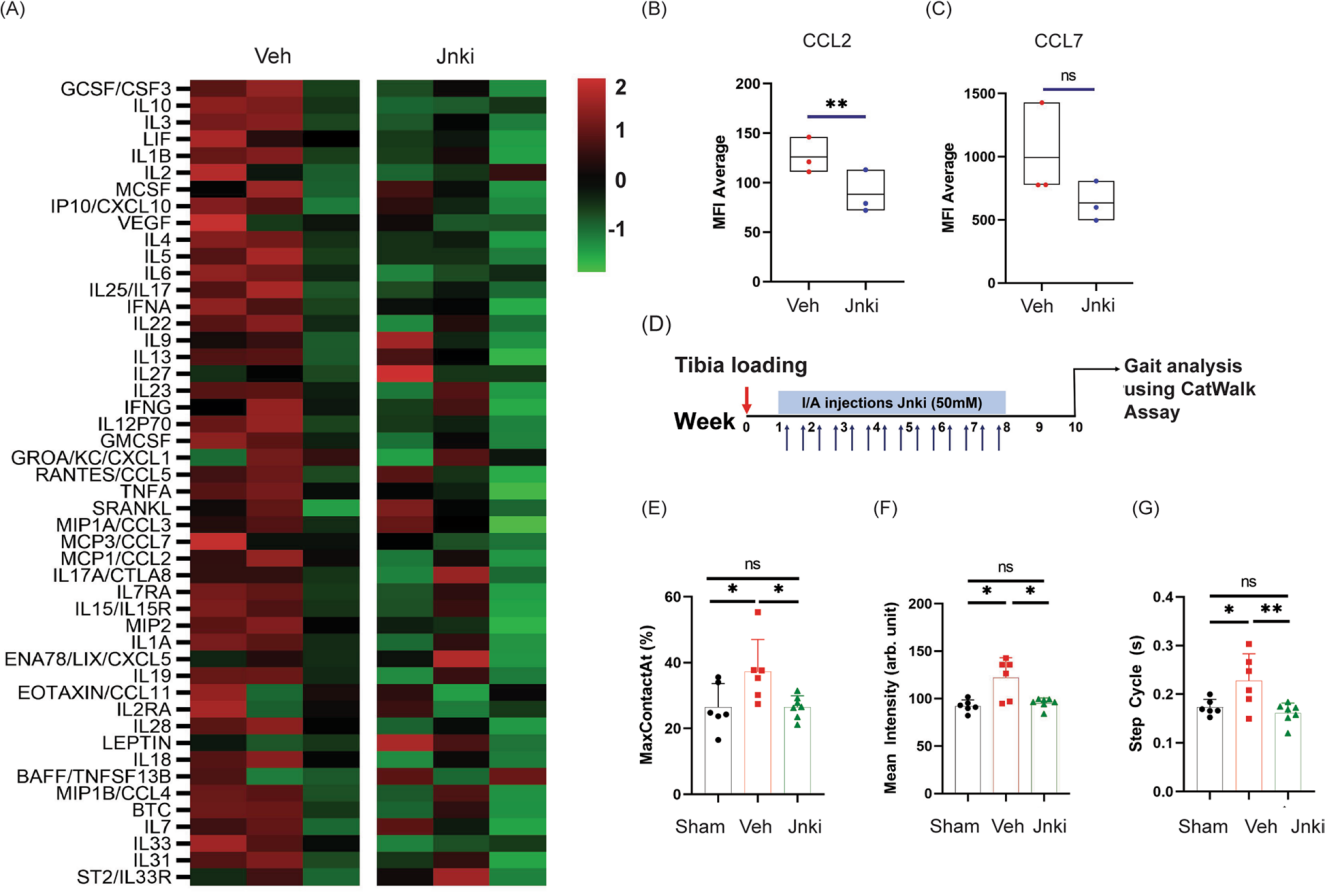
Jnk inhibition attenuates Inflammatory cytokines and improves gait in mice. **A** Heatmap showing levels of inflammatory cytokines in the serum of mice that received vehicle control or Jnki (*n* = 3, each group). **B**, **C** Levels of cytokines CCL2 and CCL7 upon Jnki administration in tibia-loaded mice as measured by mean fluorescence intensity (MFI) average; significance calculated using Student’s *t* test with Welch’s correction. **D** Schematic depicting the strategy for gait analysis of tibia-loaded mice upon Jnki administration. Changes in the gait parameters upon tibia loading and Jnki administration in mice. **E** MaxContactAt (%), **F** mean intensity (arbitrary units), and **G** step cycle (s) (S *n* (sham) = 6, *n* (Veh) = 6, *n* (Jnki) = 7), significance calculated using one way ANOVA with Tukey’s multiple comparison. *p < 0.05, **p < 0.01 (individual adjusted *p* values: mean intensity (Sham vs. Veh, 0.0024, Sham vs. Jnk2i, 0.9057, Veh vs. Jnk2i, 0.0042), MaxContactAt (Sham vs. Veh, 0.0446, Sham vs. Jnk2i, 0.9999, Veh vs. Jnk2i, 0.0371), step cycle (Sham vs. Veh, 0.0393, Sham vs. Jnk2i, 0.8104, Veh vs. Jnk2i, 0.0091)

Since InfA inhibition in human OA cartilage dampened inflammation, we next wanted to test whether JNK inhibition modulates the low-grade inflammation observed in PTOA mice. We isolated serum from the vehicle and JNKi-treated mice (*n* = 3 each) at 8 weeks post-tibia loading and analyzed using a 48-plex LUMINEX cytokine panel (Fig. 3A). We found that intra-articular competitive inhibition of Jnk in PTOA mouse model led to the dampening of multiple cytokines in the serum. Two cytokines that showed considerable reduction were CCL2 and CCL7 (Fig. 3B, C). These results are consistent with the human OA cartilage where JNK signaling inhibition led to decreased levels of CCL2 and CCL7 [20].

CCL2 is a well-known mediator of spinal synaptic transmission and pain in osteoarthritis; therefore, we wanted to test if inhibition of Jnk signaling in InfA populations would also affect pain in PTOA mice. To test this, we performed gait analysis in PTOA at 10 weeks post-tibia loading using the CatWalk platform (Fig. 3D).

JNKi-treated mice showed significant improvement in gait parameters compared to vehicle-treated controls, including Max contact (Fig. 3E), mean intensity (Fig. 3F), and step cycle (Fig. 3G). These data suggest that JNKi treatment leads to alleviation of pain and subsequent gait improvement in PTOA mice even in the absence of any structure preservation of joint.

### Intra-articular JNK inhibition does not lead to an increase in senescent cells

**Fig. 4:**
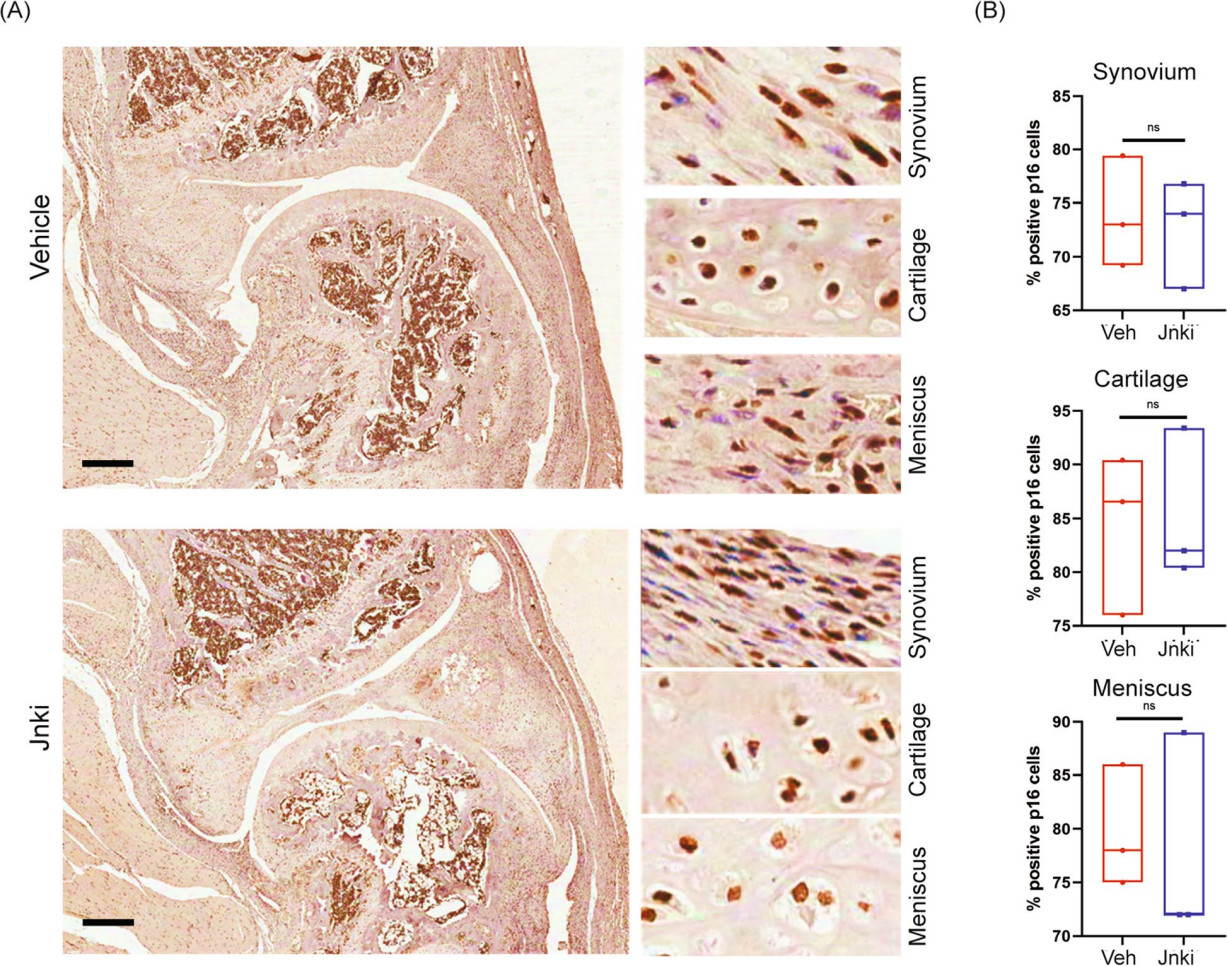
Intra-articular JNK inhibition does not lead to any increase in p16^+^ senescent cells. **A** Knee joint sagittal sections stained with p16 antibody upon 10 weeks post-tibia loading (vehicle—above, Jnki—below) showing expression of p16 in synovium, cartilage, and meniscus along with a higher magnification. **B** Quantification of p16-positive cells in synovium, cartilage, and meniscus of the tibia-loaded mice. Statistical analysis was performed using Student’s* t* test with Welch’s correction. Scale bar = 300 μm

Recent studies in JNK knockout mice showed an increase in senescence and P16 expressing senescent populations in the joints of these mice [32, 33]. We therefore investigated whether intra-articular administration of Jnki has any effect on senescent cells in the treated joints. To test this, we performed immunohistochemistry for p16, on the sagittal cross sections of tibia-loaded PTOA limbs that were treated intra-articularly with vehicle or Jnki. We were able to identify P16-expressing senescent cells induced by injury, as previously reported, in cartilage, synovium, and meniscal tissues (Fig. 4A). There was however no difference in the frequency of p16-expressing populations in cartilage, synovium, or meniscus between vehicle and Jnki treatments (Fig. 4B), indicating that the short-term, intra-articular JNKi treatment does not have an effect on the P16 expressing senescent populations.

### JNKi treatment is not chondroprotective in human OA cartilage

**Fig. 5:**
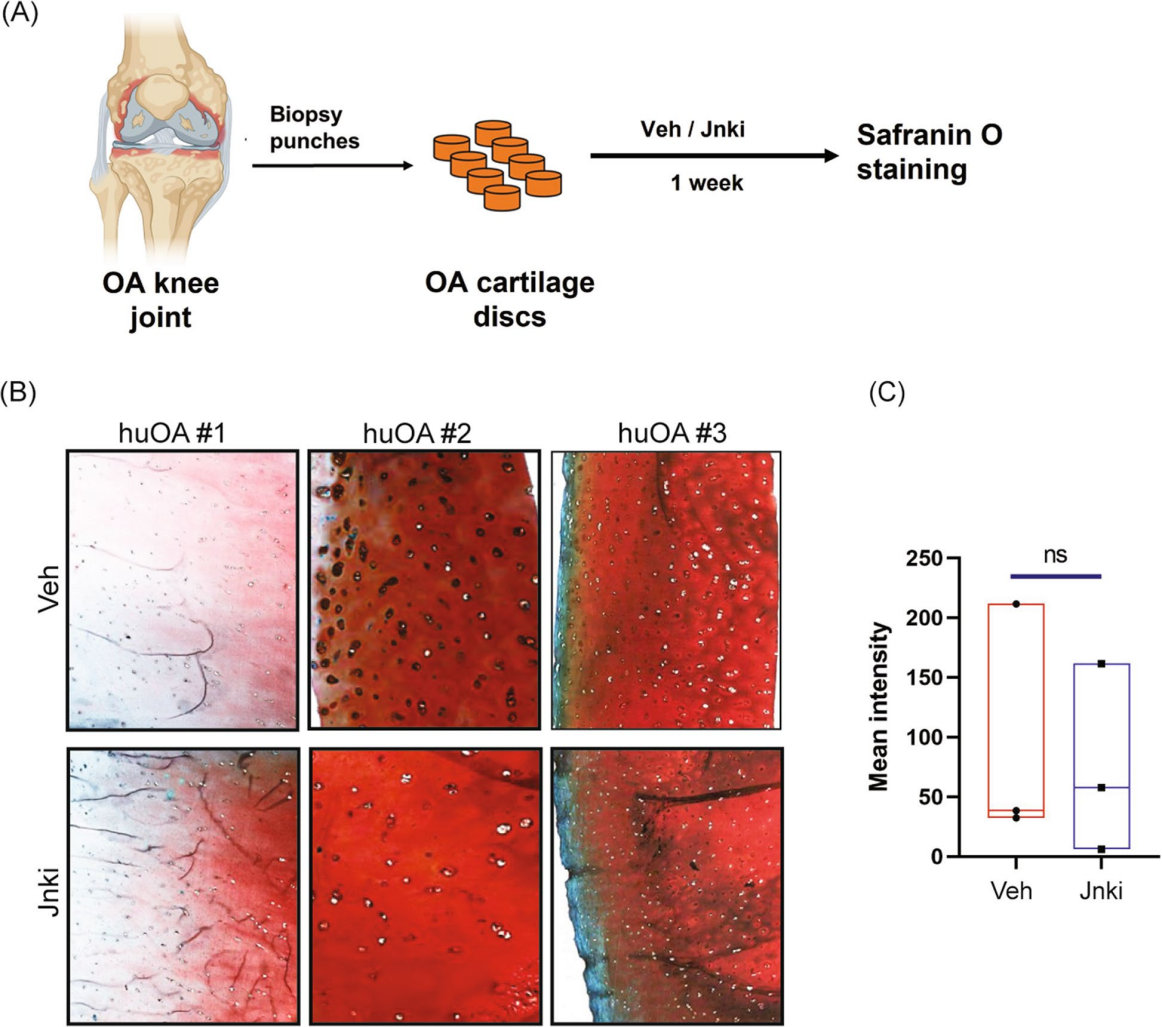
Effect of JNK inhibition on human OA cartilage. **A** Schematic showing isolation of OA cartilage discs followed by treatment with vehicle or Jnki. **B** Safranin O staining of human OA cartilage sections treated with vehicle or Jnki. **C** Quantification of Safranin O staining using ImageJ. Significance calculated using Student’s *t* test with Welch’s correction

Next, we investigated the effect of inhibition of Jnk signaling in human osteoarthritic cartilage. For this, human OA cartilage was isolated from surgical discards of multiple patients undergoing total knee replacement (under IRB protocols approved by Stanford University). Discarded cartilage from three different patients (60, 67, and 87 years old) were utilized. Uniform circular discs were generated from the patients’ OA cartilage using 4.0-mm biopsy punches and were treated with vehicle or JNKi for a week (Fig. 5A). The cartilage discs were then sectioned and stained with safranin O that stains proteoglycan (GAG) content. There was no significant difference in the intensity of the GAG content upon vehicle or JNKi treatment in OA cartilage isolated from three different patients (Fig. 5B, C). These data demonstrated that inhibition of JNK signaling does not improve cartilage structure or regeneration, similar to the effects observed in PTOA mice joints, in the human OA cartilage discs that received Jnk inhibitor.

## Discussion

Identification of precise subpopulations associated with disease pathogenesis in osteoarthritis is important for an increased understanding of the cellular basis of disease as well as for devising effective targeted therapeutics for OA. Multiple studies have identified senescent cells in aged tissues to be one such population that has deleterious paracrine effects in OA and are now being investigated for a beneficial effect in clinical trials in OA patients. Our previous single-cell studies using mass spectrometry had identified a rare IL1R1^+^TNF-R2^+^ inflammation-amplifying (InfA) population in end-stage human OA cartilage. In the present study, we have demonstrated the presence of these IL1R1^+^TNF-R2^+^ InfA cells with active JNK signaling in both aged and PTOA mice. In aged mice, Inf-A subpopulation is only present in the CD44^+^ chondrocyte compartment while the non-chondrogenic CD44^−^ cells did not show the presence of Inf-A cells, demonstrating that this is a chondrogenic cartilage-resident population comprising of about 0.1–3% of the total cell population present in the knee joint of mice. This frequency is consistent with the abundance of Inf-A in human OA cartilage as reported previously.

To test the effects of inhibiting InfA, we used a known JNK inhibitor that had been previously validated. Utilizing this JNKi, we were able to observe a lowering of CCL2 and CCL7 levels in the sera of treated mice, which was consistent with the observations in human OA cartilage where JNKi treatment led to a decrease in these very cytokines in the cartilage secretome. The CCL2/7-CCR2 molecular axis has already been implicated in the initiation and sensitization of pain in the joint CCR2 null mice being resistant to CCL2-mediated knee hyperalgesia in PTOA mice [34]. CCL2 and CCL7 are therefore interesting targets for OA pain, with CCL2 levels observed to be heightened in OA synovial fluid and correlated with pain in patient-reported outcomes (PROs) as well. Upon performing gait analysis on mice, the JNKi-treated mice showed a reduction in pain-associated behavior in multiple parameters. The maximum intensity and contact of the paw as well as the step cycle were restored back to the levels of the uninjured mice in the JNKi-treated PTOA mice. JNKi treatment therefore attenuates OA-associated pain by lowering the CCL2 levels secreted by the InfA cells. Our study therefore demonstrates that a cartilage resident population that emerges and expands with aging and injury is a major source of the pain mediator CCL2 and can be targeted specifically to modulate OA-associated pain.

Interestingly, inhibition of the InfA population through JNKi has no discernible effect on cartilage or joint homeostasis and there is no chondroprotective or joint preservation effect. Neither cartilage degeneration nor synovial hyperplasia is affected by the JNKi treatment. Consistent with mice data, no changes were observed in human OA cartilage homeostasis upon JNKi treatment assessed by GAG production and histology. A similar observation was provided by the recent report on Jnk1^−/−^Jnk2^−/−^ double knockout mice wherein no effect was observed on disease progression in a PTOA model [32]. The role of JNK signaling in OA pathogenesis has been widely studied, especially in the context of its activation and degradation of cartilage by regulating the expression of MMPs and interleukins. Recently, a study concluded that Jnk1^−/−^Jnk2^−/−^ double knockout mice did not show a difference in disease progression in a PTOA model; however, the knockout mice exhibited significantly worse cartilage in aged mice. Furthermore, the group showed that p16 positive senescent cells were in greater abundance in the Jnk1^−/−^Jnk2^−/−^ double knock-out mice as compared to the wild-type mice [32]. We therefore checked whether a short-term, intra-articular treatment with JNK inhibitor would have any effect on p16 expressing senescent cell population in the mice joints. The JNKi treatment and dosage used did not result in any increase in p16 expressing senescent cells in any of the joint tissues tested—cartilage, synovium, or meniscus.

## Conclusions

Collectively the data show that InfA cells in OA cartilage specifically regulate joint pain through the production of CCL2 and CCL7 while these cytokines have little effect on cartilage degeneration or synovial inflammation in the end-stage OA joint. It will be interesting to understand the cell populations in joint tissues that secrete other molecular mediators that are known to contribute to OA-associated pain like NGF [35, 36]. and damage-associated molecular products (DAMPs). Identification of the specific cell populations that generate these pain mediators can enhance our understanding of and provide new routes to attenuate OA-associated pain.

### Supplementary Information

**Additional file 1:** **Supplemental methods**

**Additional file 2:** **Figure S1.** Representative sagittal sections of knee joint meniscus (A) and synovium (B) from tibia loaded and control limbs showing presence of p-Jnk+ cells (InfA cells) in the cartilage, quantification in the bar graph on the right. Significance was calculated using Student t test with Welch’s correction. *p<0.05, scale bar =100μm. (C) Optimization of p-Jnk antibody on mouse knee joint sections (coronal orientation). Scale bar = 300μm

## Data Availability

All data generated or analyzed during this study are included in this published article [and its supplementary information files].

## Abbreviations

OA: Osteoarthritis
PTOA: Post-traumatic osteoarthritis
Jnk: C-Jun N-terminal Kinase
IL1R1: Interleukin 1 receptor 1
TNF-R2: Tumor necrosis factor receptor II
DMOAD: Disease-modifying osteoarthritis drug
SASP: Senescence-associated secreted proteins
Inf-A: Inflammation-amplifying chondrocytes
Inf-D: Inflammation-dampening chondrocytes
CCL2: Chemokine ligand 2
CCL7: Chemokine ligand 7
FACS: Flow cytometry-assisted cell sorting
GAG: Glycosaminoglycans
CD44: Cluster of differentiation 44
NGF: Nerve growth factor
DAMP: Damage-associated molecular pattern
P16: P16INK4a, cyclin-dependent kinase inhibitor 2A
