# Supplementary material for: Targeting an inflammation-amplifying cell population can attenuate osteoarthritis-associated pain
Source: Arthritis Res Ther. 2024 Feb 17;26:53. doi: 10.1186/s13075-024-03284-y (PMC10874031; doi:10.1186/s13075-024-03284-y)
Supplement: Supplementary file 1 — Additional file 1: Supplemental methods [file 13075_2024_3284_MOESM1_ESM.docx]

**Supplemental Methods**

**Animal procedures**

All animal procedures were approved by the Stanford University Administrative Panel on Laboratory Animal Care (APLAC 27507). Tibia loading was performed on 3-4 months old male C57BL/6 mice per published methodology^24–27^. The weight of the individual mice at the time of tibia loading was 25-30 g. After anesthesia using 2.0% isoflurane, the knee joint of the right limb was restrained in the tibia loading clamp and incremental vertical load was applied to the restrained limb until failure. Mice were intraarticularly administered a competitive inhibitor against Jnk (SP600125, 50mM in DMSO), starting 1 week post tibia loading. We chose a higher concentration as compared to previously reports^29,30^ that systemically administered the drug at 15-20 mg/kg body weight concentration to (i) enable an efficient diffusion of drug to cartilage cells - cartilage is composed of dense ECM matrix and much fewer cells when compared to other tissues and (ii) intraarticularly delivered drugs do not reside in the joint space for very long. The mice were then sacrificed after 8 or 10 weeks (to evaluate progression to OA).

**Isolation of InfA population from young and old mice**

Wild type mice at the ages 3 or 12 months were euthanized using carbon dioxide followed by cervical dislocation. Lower limbs were collected, and the foot was removed before further processing. Lower limbs were cleaned to remove skin and muscles followed by separation of knee joints from the limb. The resulting joint tissue was chopped into smaller pieces using a blade and then enzymatically digested with 0.22% collagenase digestion solution for 1 hour at 37°C with shaking. The digest was filtered through 70 μm filters to obtain single cells. The red blood cells in the resulting single cells were lysed using ACK lysis buffer. The cells were stained with TNF-R2 and IL1R1 antibodies and populations were analyzed in BD Aria FACS.

**Assessment of inflammatory cytokines**

Inflammatory cytokines were examined in the serum of mice that went under tibia loading procedure received competitive inhibitor against Jnk. Mice (n=3) were retro-orbitally bled before euthanasia. Collected blood was allowed to clot and later centrifuged at 2000g for 10 minutes at 4°C . The Mice serum samples were analyzed using LUMINEX 48-plex at the Human Immune Monitoring Center, Stanford University.

**Gait Analysis**

CatWalk system from Nodulus was utilized to investigate differences in the gait of tibia loaded mice. This system consists of an illuminating walkway that is pressure sensitive. Every step that the animal takes on the walkway gets illuminated in proportion to the pressure applied by the paw being used. This illumination of the walkway is recorded by a camera placed underneath. These recordings can be analyzed later to assess the gait of the mouse.

Mice were allowed to get accustomed to the equipment room by leaving them in the room for 30 minutes before the mice were walked on the equipment. Three compliant runs were used to assess differences between the mice. Parameters like Print area (total print area (sq mm) is the accumulated picture of the entire time the paw came in contact with the ground), MaxcontactAt (Value (%) indicates the time during a stance that max contact is obtained. This tells us which paws are used faster upon contact with the walkway, Swing (Duration of paw in the air (between stands) in seconds. This tells us which paws were raised above the ground for longer duration) etc. were determined.
