# Supplementary figures and images for: Targeting an inflammation-amplifying cell population can attenuate osteoarthritis-associated pain
Source: Arthritis Res Ther. 2024 Feb 17;26:53. doi: 10.1186/s13075-024-03284-y (PMC10874031; doi:10.1186/s13075-024-03284-y)

(A)

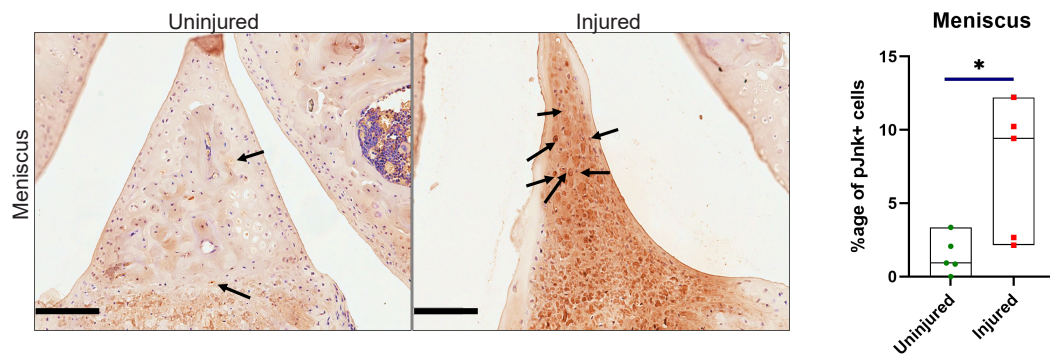

(B)

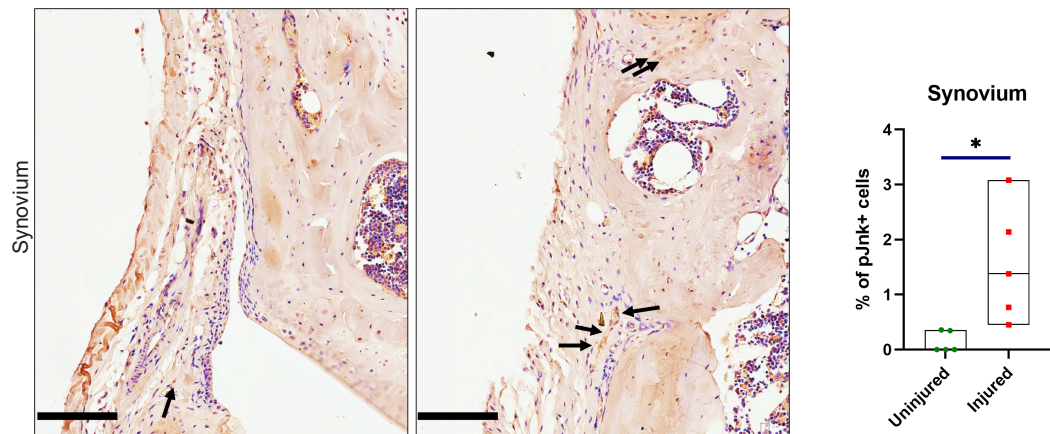

(C)

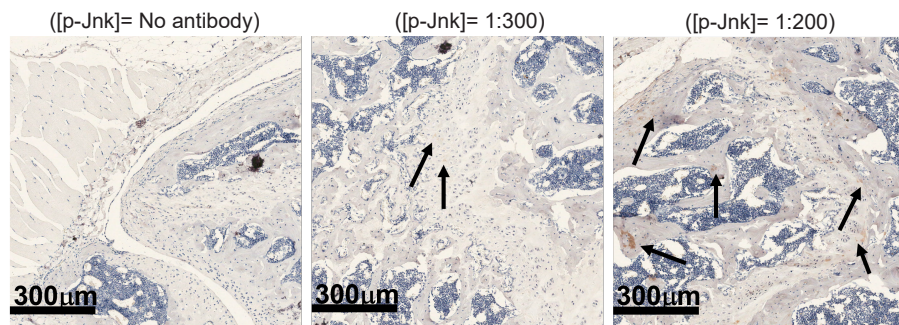

Supplement: Supplementary file 2 — Additional file 2: Figure S1. Representative sagittal sections of knee joint meniscus (A) and synovium (B) from tibia loaded and control limbs showing presence of p-Jnk+ cells (InfA cells) in the cartilage, quantification in the bar graph on the right. Significance was calculated using Student t test with Welch’s correction. *p<0.05, scale bar =100μm. (C) Optimization of p-Jnk antibody on mouse knee joint sections (coronal orientation). Scale bar = 300μm [file 13075_2024_3284_MOESM2_ESM.pdf]
